# Fatal attraction

**DOI:** 10.7554/eLife.05259

**Published:** 2014-11-25

**Authors:** Niels Ringstad

**Affiliations:** 1 **Niels Ringstad** is in the Skirball Institute of Biomolecular Medicine, Molecular Neurobiology Program and the Department of Cell Biology, NYU Langone Medical Center, New York, United StatesNiels.Ringstad@med.nyu.edu

**Keywords:** *Pristionchus pacificus*, necromeny, insect pheromones, lipid binding protein, chemosensation, nematode, *C. elegans*

## Abstract

A beetle pheromone that lures nematode worms to an insect host can also stop their development or even kill them outright.

**Related research article** Cinkornpumin JK, Wisidagama DR, Rapoport V, Go JL, Dieterich C, Wang X, Sommer RJ, Hong RL. 2014. A host beetle pheromone regulates development and behavior in the nematode *Pristionchus pacificus*. *eLife* **3**:e03229. doi: 10.7554/eLife.03229

**Image** Some nematodes are ‘necromenic’ and live inside a host and wait to feed from its carcass after it dies

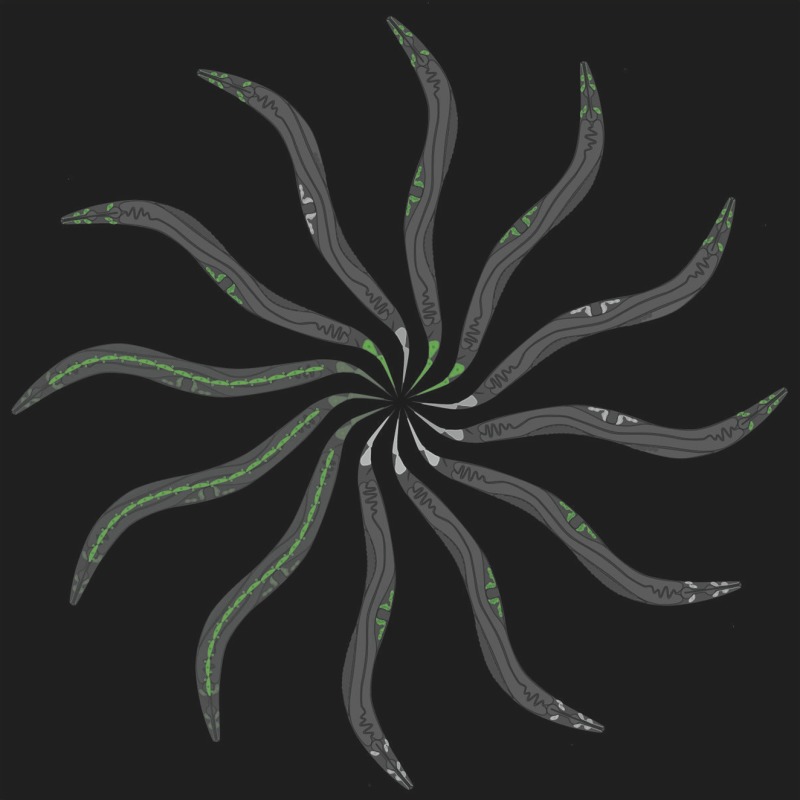

Look carefully at a solitary animal and you will find that it is not so alone after all. Animals play host to entire ecosystems that teem with diverse life. Some of the microbes that live on (or in) animals are beneficial to their host. However, these microbes' more sinister brethren, parasites and pathogens, cause damage and disease. Between these two extremes is a class of organisms that, it seems, do not harm or benefit their hosts: instead, these organisms reap their reward when the host animal dies of other causes. This lifestyle is termed ‘necromeny’ (Sudhaus and Schulte, 1989) and has been considered an evolutionary intermediate to full-blown parasitism (Sudhaus, 2008).

Now, in *eLife*, Ray Hong of California State University (CalState) and co-workers, who include Jessica Cinkornpumin and Dona Wisidagama as joint first authors, report the discovery of a molecular mechanism used by a nematode worm called *Pristionchus pacificus* to find its insect host, the oriental beetle. This necromenic nematode lives inside the beetle and waits for the beetle to die, so that it can feed off the bacteria that grow on the decomposing carcass. Working with colleagues from two Max Planck Institutes—the MPI for Biology of Ageing and the MPI for Developmental Biology—the CalState researchers have identified a new molecular player in detection of chemical signals. They have also revealed the dual nature of the chemical cue that lures these nematodes to the beetles and, at the same time, arrests their development (Cinkornpumin et al., 2014).

To understand how *P. pacificus* detects host odours, the researchers performed a genetic screen for mutant nematodes that were no longer attracted to a beetle pheromone called ZTDO. First discovered as a beetle sex pheromone, this chemical was subsequently identified as an odour that attracts *P. pacificus* (Herrmann et al., 2007). The screen identified worms with mutations in a gene called *obi-1*. This gene encodes a protein from a family of proteins that are released by diverse nematode species and bind to fatty molecules (lipids). Cinkornpumin, Wisidagama et al. speculate that the OBI-1 protein might function as a part of an extracellular clearance mechanism for lipid odorants. Such a mechanism might be required to detect small changes in odorant concentration and navigate towards the source of the pheromone.

Alternately, OBI-1 might function as part of a receptor mechanism in which a chemical cue is first bound to a protein, which then carries the signal to a receptor protein and activates it. Such multi-part odorant receptors are important for the detection of chemicals by bacteria and are also used by insects to detect some odours (Vosshall and Stocker, 2007; Kirby, 2009). Cinkornpumin, Wisidagama et al. suggest that it is possible that OBI-1 is part of a similar mechanism in the nematode.

Genetic studies of odour detection in nematodes have led to the discovery of many mechanisms behind sensory signalling that are widely conserved among animal species (Bargmann, 2006). Furthermore, uncovering factors that help to guide specific nematodes to their hosts could lead the way to new solutions to a pressing problem. Many nematodes are parasites that cause billions of dollars of damage to agricultural crops every year and levy an even more devastating toll on human health, especially in developing countries. Parasitic nematodes rely on following chemical cues to find their hosts (Chaisson and Hallem, 2012). As such, a deeper understanding of this process will offer new opportunities to develop strategies that control or eradicate populations of nematode pests.

Furthermore, Cinkornpumin, Wisidagama et al. discovered another, darker, side to the beetle's pheromone. As well as luring *P. pacificus* to a beetle, ZTDO also stops the development of the nematode or kills it outright. Thus, the very cue that *P. pacificus* uses to find its host might be used by that host to keep *P. pacificus* in check.

This other function of ZTDO in nematode-host interactions raises fascinating questions about the evolutionary origins both of the ZTDO/OBI-1 system and the origins of the necromenic lifestyle it supports. What was the ancestral function of ZTDO? Was it first used as a chemical defence against nematodes and subsequently adapted for use as a pheromone? And, if so, does this suggest that the interactions between the ancestors of *P. pacificus* and their beetle hosts were less benign than the interactions we observe today? These new insights into the mechanisms used by *P. pacificus* to find hosts suggest that necromeny might not be a way-station on the road to parasitism but might rather be a détente (or compromise) reached in the evolutionary struggle between a parasite and its host.

Many ecologically important interactions between species are observed in nature, but our understanding of how and why they happen is woefully incomplete. Now that this problem has been brought into the domain of molecular genetics through the *P. pacificus* model, we can anticipate new insights into the remarkable biology of interspecies chemical signalling.

## References

[bib1] Bargmann CI 2006 Comparative chemosensation from receptors to ecology. Nature 444:295–301. doi: 10.1038/nature05402. 17108953

[bib2] Chaisson KE Hallem EA 2012 Chemosensory behaviors of parasites. Trends in Parasitology 28:427–436. doi: 10.1016/j.pt.2012.07.004. 22921895 PMC5663455

[bib3] Cinkornpumin JK Wisidagama DR Rapoport V Go JL Dieterich C Wang X Sommer RJ Hong RL 2014 A host beetle pheromone regulates development and behavior in the nematode *Pristionchus pacificus*. eLife 3:e03229. doi: 10.7554/eLife.03229. PMC4270288 25317948

[bib4] Herrmann M Mayer WE Hong RL Kienle S Minasaki R Sommer RJ 2007 The nematode *Pristionchus pacificus* (Nematoda: Diplogastridae) is associated with the oriental beetle *Exomala orientalis* (Coleoptera: Scarabaeidae) in Japan. Zoological Science 24:883–889. doi: 10.2108/zsj.24.883. 17960992

[bib5] Kirby JR 2009 Chemotaxis-like regulatory systems: unique roles in diverse bacteria. Annual Review of Microbiology 63:45–59. doi: 10.1146/annurev.micro.091208.073221. 19379070

[bib7] Sudhaus W 2008 Evolution of insect parasitism in rhabditid and diplogastrid nematodes. In: Makarov SE Dimitrijevic RN, editors. Advances in Arachnology and Developmental Biology. Belgrade: Institute of Zoology p143–161.

[bib6] Sudhaus W Schulte F 1989 *Rhabditis* (*Rhabditis*) *necromena* sp. n. (Nematoda: Rhabditidae) from South Australian diplopoda with notes on its siblings *R. myriophila* Poinar, 1986 and *R. caulleryi* Maupas, 1919. Nematologica 35:15–24. doi: 10.1163/002825989X00025.

[bib8] Vosshall LB Stocker RF 2007 Molecular architecture of smell and taste in *Drosophila*. Annual Review of Neuroscience 30:505–533. doi: 10.1146/annurev.neuro.30.051606.094306. 17506643

